# Formation mechanism of high-index faceted Pt-Bi alloy nanoparticles by evaporation-induced growth from metal salts

**DOI:** 10.1038/s41467-023-39458-6

**Published:** 2023-06-24

**Authors:** Kunmo Koo, Bo Shen, Sung-Il Baik, Zugang Mao, Paul J. M. Smeets, Ivan Cheuk, Kun He, Roberto dos Reis, Liliang Huang, Zihao Ye, Xiaobing Hu, Chad A. Mirkin, Vinayak P. Dravid

**Affiliations:** 1 Department of Materials Science and Engineering, Northwestern University, Evanston, IL 60208 USA; 2 The NUANCE Center, Northwestern University, Evanston, IL 60208 USA; 3 Department of Chemistry, Northwestern University, Evanston, IL 60208 USA; 4 International Institute of Nanotechnology, Northwestern University, Evanston, IL 60208 USA; 5 Northwestern University Center for Atom-Probe Tomography (NUCAPT), Evanston, IL 60208 USA; 6 Department of Mechanical Engineering, Northwestern University, Evanston, IL 60208 USA

**Keywords:** Nanoscale materials, Chemistry, Design, synthesis and processing

## Abstract

Nanoparticles with high-index facets are intriguing because such facets can lend the structure useful functionality, including enhanced catalytic performance and wide-ranging optical tunability. Ligand-free solid-state syntheses of high index-facet nanoparticles, through an alloying-dealloying process with foreign volatile metals, are attractive owing to their materials generality and high yields. However, the role of foreign atoms in stabilizing the high-index facets and the dynamic nature of the transformation including the coarsening and facet regulation process are still poorly understood. Herein, the transformation of Pt salts to spherical seeds and then to tetrahexahedra, is studied in situ via gas-cell transmission electron microscopy. The dynamic behaviors of the alloying and dealloying process, which involves the coarsening of nanoparticles and consequent facet regulation stage are captured in the real time with a nanoscale spatial resolution. Based on additional direct evidence obtained using atom probe tomography and density functional theory calculations, the underlying mechanisms of the alloying-dealloying process are uncovered, which will facilitate broader explorations of high-index facet nanoparticle synthesis.

## Abstract (web summary)

Faceted nanoparticles with high-index planes exposed usually bear promising material functionalities. Here, the authors directly observe the coarsening and facet regulation process of tetrahexahedra Pt nanoparticles by using in-situ gas-phase TEM.

## Introduction

High-index-facet nanoparticles with complex shapes have unusual properties that can make them attractive for catalysis^1^, optics^2^ and magnetics^3^. For example, tetrahexahedral (THH) Pt nanoparticles exhibit excellent activity as catalysts in formic acid oxidation compared with their low-index-facet counterparts, due, in part, to the high density of surface atoms with low coordination numbers^4^. The conventional syntheses of high-index-facet particles usually require complex kinetic control processes^5–9^. Recently, a high-yield, element-general, ligand-free solid-state synthetic method was developed, which relies on an alloying-dealloying process^10–13^. In this process, a key step involves volatile foreign metals (e.g., Bi, Sb, or Pb) that initially alloy with spherical particle seeds, and then upon dealloying, stabilize the high-index-facet tetrahedra. However, relatively little is known about the transformation with respect to the coarsening dynamics, facet regulation, and foreign metal atom distribution within the resulting structures. Indeed, being able to directly visualize the process at high spatiotemporal resolution is essential to fully understand the alloying-dealloying process.

Herein, we use in situ gas-cell transmission electron microscopy (GC-TEM) to explore the formation and shape evolution of Pt nanoparticles from H_2_PtCl_6_ in the presence of trace Bi. Importantly, the GC-TEM provides a wider collection angle for high-angle annular dark field (HAADF) imaging in the scanning TEM (STEM) mode, compared to conventional environmental TEM^14–19^, and also permits the observation of reactions with a pressure up to several bars in a confined volume (20 nL); this setup is essential for accurately studying the evaporation behavior of high-vapor pressure metals, such as Bi^20,21^. With electron-transparent silicon nitride windows and a ceramic heater electrode, the synthesis of Pt nanoparticles from the initial salt precursors to their THH form was monitored under controlled environments in real time. Moreover, localized atom-probe tomography and theoretical calculations were used to understand the coarsening and facet development processes. Our findings uncover the dynamics of particle coarsening and facet regulations process in a discontinuous system, and the roles of Bi atoms in this solid-state reaction process. These nano- and atomic-scale insights will enhance the understanding of the formation mechanisms of the high-index-facet nanoparticles achieved by volatile elements and facilitate the broader explorations of these methods to other metallic systems^10,13^. In addition, this work reveals insights into the real-time pathway evolutions of nanoscale chemical reactions, with important implications for many other gas-related reactions.

## Results

### In situ synthesis of THH Pt nanoparticles

**Fig. 1:**
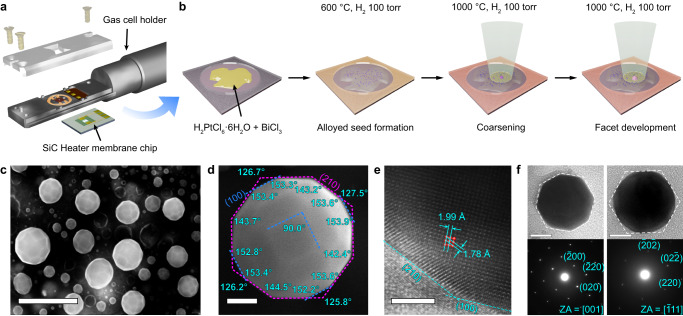
Synthesis of tetrahexahedral (THH) Pt nanoparticles using an alloying-dealloying method. **a** Schematic of the in situ gas-cell holder. **b** Schematic showing the general synthesis process of THH Pt nanoparticles with starting materials containing Bi/Pt salt precursors. **c** Scanning electron microscopy (SEM) image of the synthesized THH Pt nanoparticles. Scale bar = 500 nm. **d** Backscattered electron image of a single THH Pt nanoparticle obtained in STEM mode. This nanoparticle is tilted approximately along the [100] direction. The magenta dotted line indicates (210) facets, and the blue line indicates the (100) facets. Angles in cyan color indicate the measured interior angle of projected shape. Scale bar = 50 nm. **e** High-resolution transmission electron microscopy (HRTEM) image showing the facets of the THH Pt nanoparticle. Red dots indicate the lattice point and solid cyan lines indicate their spacings. Scale bar = 2 nm. **f** Bright-field image in TEM mode and corresponding electron diffraction patterns of THH nanoparticles, viewed along the [100], and [$\bar{1}$11] directions. White dashed lines indicate ideal THH shape projection. Scale bars = 20 nm.

A solid-state synthesis of THH Pt nanoparticles involves two stages: alloying and dealloying^10^. In the alloying stage, solid Pt nanoparticles or a metal salt act as the metal precursors. In a typical in situ GC-TEM experiment, mixtures of the Pt-containing salt precursor chloroplatinic hexahydrate (H_2_PtCl_6_·6H_2_O) and bismuth (III) chloride (BiCl_3_) were initially heated under a reductive environment to ≈600 °C to yield metal pseudo-spherical particle seeds (Fig. 1a, b, Supplementary Figs. 1–4, and Supplementary Movie 1). As the metal precursors are reduced, Bi diffuses into the Pt nanoparticles, and alloys were formed. The gas cell was then heated to 1000 °C to initiate the dealloying stage.

In this process, the nanoparticles grow via coalescence, and distinct facets appear (Supplementary Fig. 2c). Most of the generated nanoparticles have a slightly truncated THH shape, a Wulff reconstruction of a nanoparticle solely covered by (210) planes (Fig. 1c, d). The THH shape can be described by six square pyramids along the {100} planes of the regular cube with the height of each pyramid ½ of the edge of the base cube. The truncation of the nanoparticles occurs at the apex of the pyramids with (100) planes exposed (Fig. 1e). Along the [100] direction, the projection of the synthesized nanoparticles exhibits an octagonal shape, while, along the [$\bar{1}$11] direction, the THH-shaped nanoparticles display a regular hexagonal feature with a slight truncation of the (100) planes (Fig. 1f).

**Fig. 2:**
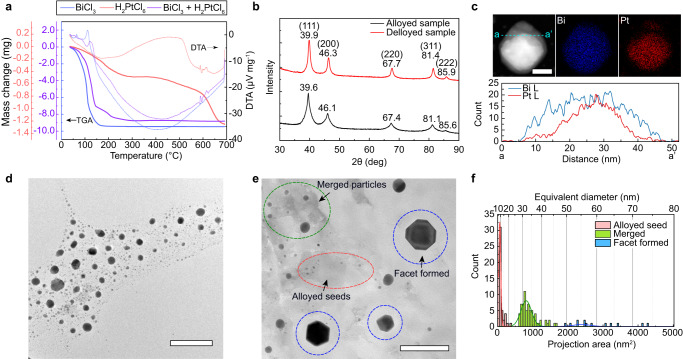
Characterization of alloyed and dealloyed Pt nanoparticles. **a** Thermogravimetric analysis (TGA)-differential thermal analysis (DTA) of the pure and mixed salt precursors as a function of temperature. Solid lines are TGA results and dashed lines are DTA results. **b** X-ray diffraction (XRD) results for the alloyed and dealloyed samples. **c** Scanning transmission electron microscopy (STEM) and elemental maps and line profiles of Bi (blue) and Pt (red) for the nanoparticles after alloying. Scale bar = 20 nm. **d** Alloyed Bi-Pt nanoparticles after heating the salt precursor to ≈600 °C. Scale bar = 200 nm. **e** Post-mortem (after the in situ experiment) TEM image of the nanoparticles formed after dealloying. Scale bar = 100 nm. **f** Statistical size distribution of the nanoparticles after dealloying. The size range of the Pt–Bi alloyed seeds is 10.07 ± 3.31 nm (*n* = 69), that of the merged nanoparticles is 33.84 ± 4.32 nm (*n* = 61), and the range of the faceted nanoparticles is 62.21 ± 12.23 nm (*n* = 14).

To understand the reactions in the alloying-dealloying process, we performed thermogravimetric analysis and differential thermal analysis (TGA-DTA) on the Pt/Bi precursors and their mixtures (Fig. 2a and Supplementary Fig. 5). The TGA-DTA data of the mixed salt precursors indicate two major exothermic reactions. The first exothermic peak is located at ≈120 °C; this peak is located ≈10 °C higher than the exothermic peak of the pure BiCl_3_ precursor, indicating the formation of Bi metal. The second exothermic peak is located at ≈580–600 °C (close to the exothermic peak of the H_2_PtCl_6_ salt), indicating the formation of Pt nanoparticles. Considering that the melting point of Bi is ≈272 °C, Pt nanoparticles can be alloyed with Bi atoms during this stage^22^. When heated to 1000 °C, a significant mass loss for the mixed precursors was observed (Supplementary Fig. 5), corresponding to the evaporation of Bi. X-ray diffraction (XRD) shows that both the alloyed and dealloyed samples can be indexed as Pt with a face-centered cubic (FCC) structure (Fig. 2b). The alloyed samples have peaks with larger full widths at half maxima, indicating that the average nanoparticle size in these samples is smaller than nanoparticles in the dealloyed samples. For the dealloyed samples, the peaks are shifted to slightly higher angles, indicating that the lattice parameter of the Pt nanoparticles in the dealloyed samples is slightly smaller (by ≈0.4%) than those in the alloyed samples. Since a Bi atom has a larger radius (≈207 pm) than a Pt atom (≈175 pm), when Bi evaporates from alloyed Pt nanoparticles, lattice contraction occurs, which agreed with the XRD results.

The individual alloyed nanoparticles were characterized by STEM energy dispersive X-ray spectroscopy (EDS), and it was determined that Bi atoms diffuse into Pt nanoparticles at ≈600 °C (Fig. 2c). The Bi distribution varies within single nanoparticles; Bi-rich and Pt-rich regions were observed. Bright-field images of the general structural features of the alloyed and dealloyed samples reveal that faceted nanoparticles form during the dealloying process (Fig. 2d, e). Moreover, the dealloyed Pt nanoparticles (formed after annealing at 1000 °C) can be classified into three categories: pristine Pt–Bi alloyed seeds, coarsened nanoparticles, and faceted nanoparticles. Most of the pristine alloyed seeds have a spherical shape with a size of 10.07 ± 3.31 nm (Fig. 2f). At high temperatures, these Pt–Bi alloyed seeds coalesce. The coarsened nanoparticles are between 20 and 50 nm in size and do not exhibit faceted features. When the nanoparticles grow larger than 50 nm, facet features were frequently identified (Fig. 2e). The faceted nanoparticles have the geometric characteristics of tetrahexahedra (Supplementary Fig. 6).

### Coarsening of Pt–Bi alloy nanoparticles

**Fig. 3:**
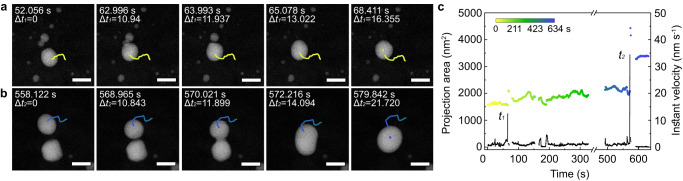
Coalescence growth kinetics for primitive Pt–Bi alloy nanoparticle seeds. **a**, **b** Time-series high-angle annular dark field (HAADF) images showing migration kinetics of two individual nanoparticles near the point of merging. Inset curves show the trajectories of the Pt–Bi alloy nanoparticles. Scale bar = 50 nm. **c** Graph displaying the instant velocity (colored) and the projection area (black) changes of the nanoparticles.

The morphology of the formed Pt–Bi alloy seeds did not change after extended annealing at 600 °C (Supplementary Fig. 3), implying that the dealloying process cannot be triggered at this temperature. TGA-DTA plots and thermal peaks further confirm that no obvious changes in mass occur between 600 and 900 °C (Supplementary Fig. 5). However, between 900 and 1000 °C, significant mass loss occurred, attributed to Bi dealloying. During the in situ GC-TEM analysis at temperatures between 900 and 1000 °C, the previously stationary Pt–Bi alloy nanoparticles vibrated or moved (Supplementary Movie 2). In one process, a nanoparticle (≈25 nm in size) moves at a speed of ≈1.05 nm s^−1^ and then at ≈12 nm s^−1^ upon coalescence with its neighbors to form larger nanoparticles (Fig. 3a, 3c). While the other smaller nanoparticles in the frame (< 10 nm) only vibrate slightly (Supplementary Fig. 7). In another sequence (Fig. 3b), the pristine alloyed Pt–Bi nanoparticles have a similar size (≈30 nm), but different morphologies. The top nanoparticle moves continuously with a speed of ≈1.1 nm s^−1^. The bottom faceted nanoparticle does not move or vibrate. At the point of coalescence, the instant velocity of the top nanoparticle spikes (≈32 nm s^−1^), and its direction changes accordingly. Upon coalescence, the nanoparticle becomes more isotropic, and its projection area is reduced (Fig. 3c). Meanwhile, the instant velocity decreases to zero and facets start forming (vide infra).

### Evaporation-induced facet development

**Fig. 4:**
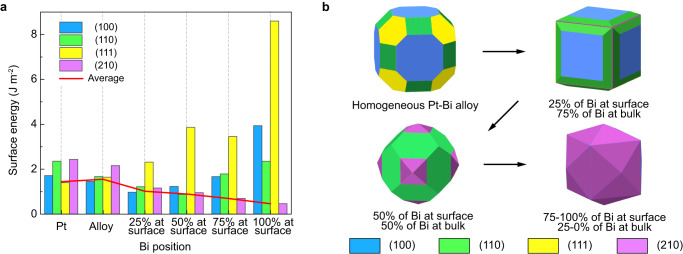
First-principle calculations of the surface energy change. **a** Surface energy changes of different facets depending on the spatial distribution of Bi in a Pt nanoparticle. **b** Wulff construction of nanoparticles with different Bi distributions.

To gain a fundamental understanding of facet evolution, we used density functional theory (DFT) calculations to simulate the surface energy change during the alloying-dealloying process (Fig. 4a). Models of pure Pt_N_ and alloyed Pt_N-n_Bi_n_ clusters were built to represent pure Pt nanoparticles and homogeneous Pt–Bi alloys, where N is the total atom numbers in the slab structure and n is the number of Bi atoms in the model. The DFT results show that the (111) facets are the most energetically stable ones in the pure Pt nanoparticles, and the corresponding Wulff construction is a truncated octahedron with an average surface energy of 1.448 J m^−2^. In the alloying process, eight Bi atoms randomly substitute for the interior Pt atoms. In the formed Pt_N-n_Bi_n_ clusters, the (100) planes have the lowest surface energies, and the corresponding Wulff construction is a truncated cuboctahedron with a higher average surface energy of 1.556 J m^−2^. This result indicates that homogeneous Bi substitution in bulk Pt is energetically unfavorable. In the dealloying process, interior Bi atoms are segregated to the surface, and the average surface energy decreases (Supplementary Table 1 and Fig. 4a), indicating that the outward diffusion of Bi is thermodynamically preferred. Moreover, the surface energy of the (210) facets decreases dramatically when more Bi atoms are on the surface, driving the evolution of the Wulff constructions (Fig. 4b). For example, an edge-truncated cube with (100) planes is the most stable with 25% Bi segregation to the surface, while a (210) planes pyramid-capped rhombic dodecahedron with (110) planes is the most stable with 50% Bi segregation to the surface. When 75% of the Bi atoms are segregated on the surface, the (210) planes become more stable than the low-index planes, resulting in a tetrahexahedron Wulff construction. As outward diffusion continues and 100% of the Bi atoms are at the surface, the average surface energy is further reduced and the (210) planes are stabilized. These calculations show that even without complete diffusion of all of the Bi atoms to the surface, the nanoparticle morphology can still evolve into the THH shape.

**Fig. 5:**
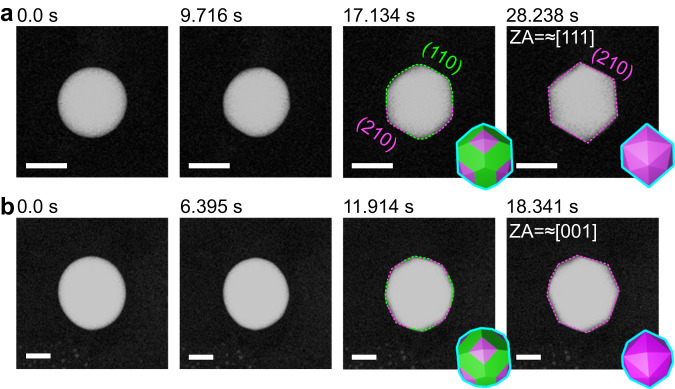
Facet development of Pt–Bi alloy nanoparticles. Time-series HAADF images showing facet development of a THH nanoparticle, viewed approximately along the **a** [111] zone axis and **b** [001] zone axis. The scale bars in (**a**, **b**) are 50 nm. Green dashed lines in the HAADF image and green faces in the inset Wulff shape indicate (110) facets. Magenta dashed lines in the HAADF image and magenta faces in the inset Wulff shape indicate (210) facets.

We correlate the above theoretical calculations to the observed facet development process captured with in situ GC-TEM (Fig. 5) The nanoparticle was isotropic in shape at first, typically composed of many localized low-index facets^23^, and then it transformed to a THH shape within ≈30 s. Primitive edges start to appear at ≈9.7 s (Fig. 5a and Supplementary Movie 3). After ≈17.1 s, the projected image of the nanoparticle is a hexagon with rounded corners. Finally, after ≈28.2 s, the corners of the hexagon become perfectly sharp and match the projected image of a THH nanoparticle along the ≈[111] direction. At this moment, at least 75% of the Bi atoms have diffused from the interior to the surface of the particle. Since there is not an abrupt rotation during this process, the round-cornered hexagon can be correlated to the Wulff shape with ≈50% Bi segregation at the nanoparticle surface. The smooth corners of the hexagon can be correlated to the projection of intermediate (110) facets that transform to sharper (210) facets later when more Bi atoms diffuse to the surface. A similar process was also observed for the nanoparticle approximately projected along the [001] direction (Fig. 5b and Supplementary Movie 4). The primitive edges start to appear ≈6.4 s after the formation of an isotropic sphere. At ≈11.9 s, the particle has a round-cornered octagonal shape, which is the projected image of the Wulff shape with 50% Bi segregation at the surface. Finally, the projected image of the nanoparticle becomes an octagon with perfectly sharp corners, the Wulff shape with over 75% Bi atoms at the surface. Here, we want to point out that the electron beam effect during the observation can be neglected since the similar faceted THH Pt nanoparticles can be also broadly observed in the electron beam non-exposed areas (Supplementary Fig. 8).

### Surface segregation of Bi atoms

Bi segregation and evaporation were proposed to rationalize the facet formation process. To validate this understanding, direct evidence of Bi segregation at the nanoparticle surface with nanoscale spatial resolution is necessary. However, it is challenging to quantify and map trace amounts of Bi on the surface of or inside nanoparticles with sizes between 100 and 500 nm. For example, secondary ion mass spectroscopy can offer highly sensitive atomic quantification throughout the shallow depth of interest, but the lateral resolution is insufficient to localize certain atoms with nanometer precision. In contrast, elemental quantification using TEM-based techniques offer high spatial precision, but it is practically impossible to detect low concentrations of heavy elements with electron energy loss spectrum (EELS). Also, the characteristic X-ray emissions of Bi Lα and Pt Lβ_1,2_ are only 232 eV apart, making elemental quantification difficult because the peaks are convoluted. Furthermore, considering that TEM only provides projected information, EDS and EELS mapping cannot distinguish the surface and interior of the nanoparticle effectively (Supplementary Fig. 9). As an alternative, atom-probe tomography (APT) can provide highly sensitive mass spectra based on elemental quantification in three-dimensional (3D) space within several hundreds of nanometers; the spatial resolution is up to 0.3 nm in depth^24,25^.

**Fig. 6:**
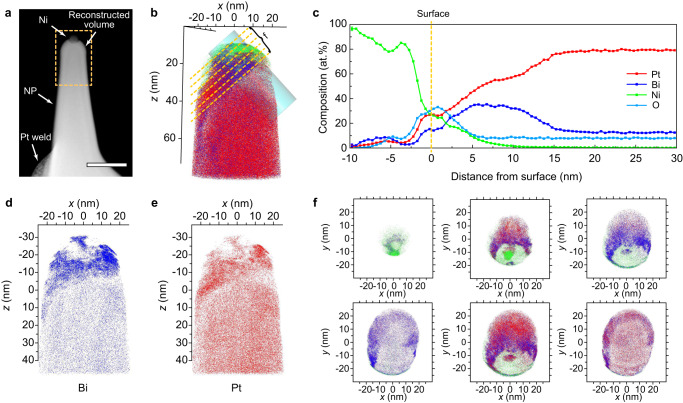
Elemental analysis of THH nanoparticles after dealloying using atom-probe tomography. **a** HAADF image of an atom-probe specimen. The yellow dashed box indicates actual APT reconstruction volume. Scale bar = 100 nm. **b** Reconstructed total atomic map with green Ni, blue Bi, and red Pt atoms. Yellow dashed lines indicate the cross-section position for (**f**). **c** Proximity histograms of elemental profiles showing composition variations from interior to surface. **d**, **e** Reconstructed Bi and Pt volume. **f** *xy* cross-sectional image normal to the Bi segregation plane. The interval between the cross-sections is 5 nm. Ni, Bi, and Pt atoms are marked with green, blue, and red color, respectively.

Therefore, we used APT to track trace amounts of Bi on the surface of the THH Pt nanoparticles (Supplementary Fig. 10); a 40 nm (*w*) × 40 nm (*d*) × 120 nm (*h*) specimen near the surface of a nanoparticle was reconstructed (Fig. 6 and Supplementary Movie 5). The correlative TEM analysis of an APT nanotip (Fig. 6a) presents the surface of a Pt nanoparticle with a nickel (Ni) protective coating layer, and a 3D APT reconstruction with ≈20 million atoms is displayed (Fig. 6b). The THH nanoparticle is composed of Pt atoms (red), visible on the right-lower side of the 3D reconstruction, and Bi atoms (blue) under a Ni (green) coating layer. For quantitative analysis, a concentration profile within a cylindrical region (diameter: 20 nm) was generated normal to a facet of the nanoparticle utilizing the proximity histogram method (Fig. 6c). The surface of the Pt nanoparticle was placed at the cross point of the Pt and Ni concentrations (marked zero on the abscissa axis). Above the top protective Ni layer, there is a 5 nm nickel oxide layer resulting from oxidization that occurred during the Ni sputter coating process. This nickel oxide layer indicates that the original surface of the nanoparticle is retained. The atomic ratio of Pt and Bi changes as a function of depth. The atomic concentration of Bi at the surface down to a depth of 11.5 nm is 40% with the other 60% being Pt and trace elements. However, the concentration of Bi decreases to 13% deeper than 11.5 nm from the surface, indicating the segregation of Bi atoms at the surface. Separate 3D reconstructions of Bi and Pt volume and *xy* cross-sectional images normal to the Bi segregation plane also indicate Bi enrichment at the surface (Fig. 6df).

To further demonstrate the role of surface segregation of Bi to the morphological change, as-synthesized THH Pt nanoparticles were washed with sulfuric acid (1 M H_2_SO_4_ for 1 h) to remove surficial Bi ex situ, and then annealed at 1000 °C for 10 min to obverse the shape change. As shown in Supplementary Fig. 11, the THH shape of Pt nanoparticles is retained at room temperature after the acid treatment. However, the following annealing leads to all (210) facets of the nanoparticles disappeared, indicating Bi segregation is important to the stabilize the THH Pt nanoparticles. Also, the surficial Bi of the THH Pt nanoparticles can be removed by a prolonged time annealing. We found that when the THH Pt nanoparticles were annealed for >3 h, Bi atoms at the Pt nanoparticle eventually depleted and the Pt nanoparticles lost the THH shape (Supplementary Fig. 12).

## Discussion

**Fig. 7:**
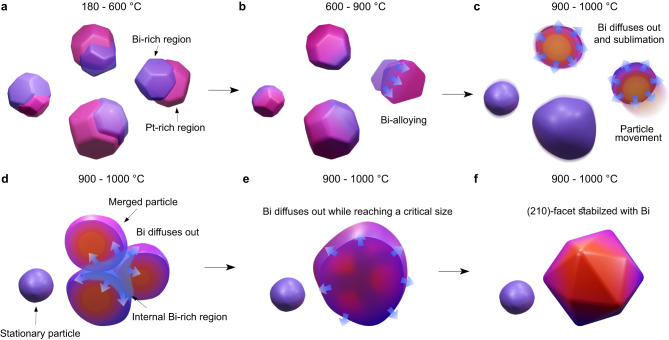
Schematic describing the evaporation-induced growth process of THH nanoparticles. **a** In the range of 180–600 °C, Bi and Pt salt precursors decompose and form nanoparticles with Bi-rich (indicated by purple) and Pt-rich (indicated by magenta) regions. **b** In the range of 600–900 °C, two metals are alloyed with each other. **c** In the range of 900–1000 °C, Bi atoms start to dealloy from the nanoparticles, and nanoparticles become mobile. **d** Nanoparticles are merging and generate a new internal Bi-rich region. Bi atoms further diffuse out from the merged particles. **e** The merging process continues until the nanoparticles reach to a critical size. Bi atoms continue to diffuse out. **f** Finally, the surficial Bi atoms stabilize (210) facets of the Pt nanoparticles, forming the THH shapes.

Based on the above experimental and theoretical results, a synthesis process for THH Pt nanoparticles was proposed (Fig. 7). Between 180 and 600 °C, the Bi precursor decomposes first due to its lower thermal stability, and subsequently, the Pt precursor decomposes at a higher temperature, forming Bi and Pt nanoparticles. Since these precursors are mixed, and Bi is a volatile element with a melting point of 272 °C, the Bi atoms evaporate and diffuse into the Pt nanoparticles to form a Pt–Bi alloy (Fig. 7a). The Bi concentrations vary for different nanoparticles or even within different regions of single nanoparticles. Between 600 and 900 °C, the Bi atoms redistribute within the alloyed Pt nanoparticles and there are slight changes in the nanoparticle morphology (Fig. 7b). Above 900 °C, the alloyed Pt nanoparticles become unstable, and the Bi atoms diffuse outward to the surface. Simultaneously, some of the alloyed Pt nanoparticles vibrate and move at around 1 nm s^−1^ (Fig. 7c). The driving force for the latter results from the evaporation of surface Bi atoms and the unbalanced total surface energy of the alloyed Pt nanoparticles. Therefore, particles can merge at this stage with neighboring ones on their same trajectory (Fig. 7d). After merging, Bi atoms that were previously on the surface can become interior atoms in the newly formed nanoparticles (Fig. 7e), and the outward diffusion of Bi continuously occurs. With Bi diffusion, nanoparticle morphology changes gradually on the 10–30 s timescale. When a localized balance state is reached, the Pt nanoparticles stop moving and vibrate in place. A spherical particle enclosed by many localized low-index planes is formed. Consequently, the Bi atoms in the spherical nanoparticles redistribute to the {210} crystallographic planes, and THH-shaped nanoparticles are formed (Fig. 7f).

In summary, we examined the evaporation-driven synthesis of high-index faceted Pt nanoparticles using in situ GC-TEM. We directly observed that the reaction dynamics of the alloying-dealloying process at the nanoscale relies on the formation of a Pt–Bi alloy at ≈600 °C. At higher temperatures (≈900–1000 °C), Bi atoms diffuse to the outer surface of the alloyed nanoparticles, and the associated Bi evaporation promotes the coarsening process and high-index-facet formation. The APT data for the dealloyed Pt samples provide direct evidence of surface Bi segregation at the atomic scale. Due to the further redistribution of surface Bi atoms on some specific facets, the Pt nanoparticles evolve into a THH shape with {210} facets, the structure which possesses the lowest surface energy according to theoretical calculations. These insights into the reaction dynamics provide direct evidence of the coarsening and facet regulation processes of Pt nanoparticles with high-index facets and will facilitate further exploration of alloying-dealloying methods in other metallic systems with diverse crystal structures (e.g., Ru, Fe, Zr).

## Methods

### Materials and bulk characterization

Chloroplatinic acid hexahydrate (H_2_PtCl_6_·6H_2_O, ACS reagent, ≥37.50% Pt basis), bismuth (III) chloride (BiCl_3_, anhydrous, powder, 99.998% trace metals basis) and hydrochloric acid (ACS reagent, 37%) were purchased from Sigma Aldrich. Millipore ultrapure water (18.2 MΩ cm) was used as the solvent for all solutions. The pH of the BiCl_3_ solution was tuned to 2 to prevent the hydrolysis of BiCl_3_.

TGA-DTA of the salt precursors and their mixtures were performed in a Netzsch STA 449 F3 Jupiter Simultaneous Thermal Analysis (STA) instrument. 0.1 mL of H_2_PtCl_6_·6H_2_O (20 mg mL^−1^), 0.1 mL of BiCl_3_ (95 mg mL^−1^), and their 1:1 mixture (v:v) were placed separately in a 0.3 mL alumina crucible and dried overnight with an indirectly illuminated infrared lamp. The samples were measured under 5% H_2_ balanced with N_2_ gas (25 mL min^−1^). The buoyancy effect for gas was corrected by measuring the empty crucible under the same measurement conditions used for the samples. The temperature was increased at a rate of 10 °C min^−1^, and the thermobalance of the STA was verified using a certified sample of calcium oxalate monohydrate (European Pharmacopoeia Reference Standard) up to 1000 °C. The XRD data were collected on a Rigaku Ultima with a Cu Kα source. SEM images were acquired with a Hitachi SU-8030 field-emission electron microscope with a voltage of 30 kV and a current of 20 μA. X-ray photoelectron spectra were acquired using the Thermo Scientific ESCALAB 250Xi system.

### In situ and ex situ TEM characterizations

Closed-cell-type gas environmental cell transmission electron microscopy was conducted using an Atmosphere 210 System (Protochips Inc.). The instrument was equipped with a specimen rod and a gas supply manifold that can flow in experimental gas up to 1 atm. The specimen rod can accommodate two windowed micro-electro-mechanical system chips. The system can supply the electrical load to one of the chips, which was deposited with a thin-film ceramic heater. The heater membrane can be heated up to 1000 °C under the desired gas conditions. Before assembling the bottom and top chips, we performed plasma treatment to remove any residual hydrocarbon contamination and enhance the wettability. 1 µL H_2_PtCl_6_·6H_2_O (1.1 mg mL^−1^) and 1 µL BiCl_3_ (0.01 mg mL^−1^) solution were drop-casted on the bottom heater membrane. The windowed chips were stacked facing each other with the fluoroelastomer gasket to make an airtight channel inside of the specimen rod. After purging the cell interior with ultrahigh purity (UHP) argon, 200 torr reductive gas (50% hydrogen and 50% argon) was flown into the closed cell at the rate of 0.1 atm cm^3^ min^−1^. Then, the membrane was heated to 600 °C to facilitate the formation of the Pt–Bi alloy seeds. After 10 min, the membrane was further heated to 1000 °C to accelerate the coarsening and dealloying process and environmental parameters, including temperatures, pressures, and gas species, were recorded and plotted (Supplementary Fig. 1).

All in situ and ex situ data, including bright-field images, HRTEM images, electron diffraction patterns, backscattered electron images, and HAADF and EDS maps, were obtained using a JEOL ARM 200CF, which was operated at 200 kV. This microscope was equipped with a field-emission gun, probe corrector, and dual silicon drift detectors.

### Dynamic tracking of nanoparticles

Drift-corrected in situ HAADF images were acquired using Protochips AXON software. Evaluations of the nanoparticle velocity, size, and shape during the coarsening process were extracted using a MATLAB code to avoid bias and error^26^. The built-in open-source functions in the MathWorks image processing toolbox were utilized^27^. The videos were split into frames, and the pixels in each frame were binarized into logic matrices since the binary morphological operation can smoothen the shade consistency of nanoparticles and background spaces. Then, each frame was converted into binary values according to the averaged cutoff threshold, with 0 being background pixels and 1 being nanoparticle pixels. Consequently, neighboring nanoparticle pixels were categorized into groups, and potential nanoparticles were defined. In addition, associated data, including the centroid, size (defined as the number of pixels), major axis length, and boundary location, were identified. After removing the background white noise and tagging the remaining groups as real nanoparticles, the size of the potential nanoparticles was then compared to a minimum size threshold to avoid unreasonable extraction. Finally, automatic tracking on the same nanoparticle was performed in each frame extracted from the videos. By defining a centroid lock on the targeted tracking nanoparticle in frame 1, the nanoparticle with the closest centroid in the next frame was calculated to be the same nanoparticle. With a loop through all frames, the actual size, location, and velocity of the target nanoparticle were extracted and stored in the result arrays. The exact nanoparticle highlighted by the green outline was plotted on each frame (Supplementary Movie 6).

### DFT calculations

DFT calculations were used in this study, employing the plane-wave total-energy methodology with the Perdew-Burke-Ernzerhof parametrization of the generalized gradient approximation (GGA)^28,29^ for exchange-correlation, as implemented in the Vienna ab initio simulation package (VASP)^30–34^. We used the projector augmented wave (PAW) potentials^35^. Unless otherwise specified, all structures were fully relaxed with respect to volume as well as all cell-internal atomic coordinates. We carefully considered and tested the convergence of results with respect to a range of energy cutoff and *k*-points. A plane-wave basis set was used with an energy cutoff of 600 eV to represent the Kohn–Sham wave functions. The summation over the Brillouin zone for the bulk structures was performed on an 8 × 8 × 8 Monkhorst-pack *k*-point mesh for all calculations. Bulk Pt_N-n_Bi_n_ with and without vacuum separation were constructed for different surface planes, where N represents the total atom number of the model structure and n represents the number of Bi atoms in the model. To simulate the alloying process, Bi atoms are randomly substituted for the interior Pt atoms. For models with 25%, 50%, 75%, and 100% surface coverages in the dealloying process, we force 25%, 50%, 75%, and 100% Bi atoms to substitute the Pt atoms on the surface. The total energies with volume constraints were calculated for these fully relaxed model structures. The surface energy, *σ*, of different planes is determined by the following:

1 $$\sigma=\left({E}_{{{{{{\rm{Surface}}}}}}}^{{{{{{\rm{bulk}}}}}}{{{{{{\rm{Pt}}}}}}}_{N-{{{{{\rm{n}}}}}}}{{{{{{\rm{Bi}}}}}}}_{{{{{{\rm{n}}}}}}}}-{E}^{{{{{{\rm{bulk}}}}}}{{{{{{\rm{Pt}}}}}}}_{N-{{{{{\rm{n}}}}}}}{{{{{{\rm{Bi}}}}}}}_{{{{{{\rm{n}}}}}}}}\right)/A$$

Where ${E}_{{{{{{\rm{Surface}}}}}}}^{{{{{{\rm{bulk}}}}}}{{{{{{\rm{Pt}}}}}}}_{N-{{{{{\rm{n}}}}}}}{{{{{{\rm{Bi}}}}}}}_{{{{{{\rm{n}}}}}}}}$ is the total bulk energy of Pt_N-n_Bi_n_ of different surface planes with 6 Å vacuum separation, ${E}^{{{{{{\rm{bulk}}}}}}{{{{{{\rm{Pt}}}}}}}_{N-{{{{{\rm{n}}}}}}}{{{{{{\rm{Bi}}}}}}}_{{{{{{\rm{n}}}}}}}}$ is the total bulk energy of Pt_N-n_Bi_n_ with no surface, and A is the area of the surface. The substituting energy of Bi in Pt bulk is 0.756 eV atom^−1^. The surface was built on an 8 × 8 × 2 slab with a 6 Å vacuum. We have found that 6 Å plane separation is sufficient to minimize the effects between separation planes. Wulff construction was used to predict the morphology of the nanoparticles^36–38^.

### Atom-probe tomography

Samples for both STEM and APT characterization were prepared by the lift-out method^39^ employing a dual-beam focused ion beam (Helios Nanolab, FEI). A wide, wedge-shaped piece (30 × 6 μm^2^) was cut from a bulk sample, which contained many Pt–Bi nanoparticles under a Ni coating layer. The samples are followed by liftout, and then attached to the modified Cu grid post utilizing an Omni-probe micromanipulator. After attaching the sample to the Cu grid, it was thinned by Ga ion beam milling with a final energy of 2 kV at 24 pA, until it was electron-transparent in the TEM (≈100 nm thickness). After STEM and EDS analyses of the TEM sample, the outer portion of a Pt–Bi nanoparticle on the thin lamellar sample was cut out and sharpened to form an APT nanotip with an ≈50 nm radius of curvature via Ga ion beam through annular milling. The final nanotip samples were transferred to the local-electrode atom probe (LEAP).

A high detection efficiency (≈80%) LEAP tomograph (LEAP 5000XS, Cameca, Madison, WI, USA) was utilized to measure the chemical compositions of the Pt–Bi nanoparticle in the nanotip sample. A sharpened APT nanotip on a TEM grid was loaded into the LEAP tomograph immediately after TEM characterization. Picosecond pulses of UV laser light (355 nm) were applied to evaporate individual atoms from APT nanotips with a pulse repetition rate of 250 kHz at 40.0 ± 0.3 K. The laser energy was 40 pJ pulse^−1^, and the average detection rate was 0.005 ions pulse^−1^. The nanotips were maintained at a positive potential, while the evaporation of ions was triggered by the picosecond UV laser pulses. The times-of-flight of the detected ions were utilized to produce 3D reconstructions using the tip profile method in the software IVAS with a version of 3.8.8. The proximity histogram methodology was utilized to study the compositional variations within the nanoparticle^25,40^ (see Supplementary Fig. 13 for a full-range mass spectrum of the APT tip).

### Reporting summary

Further information on research design is available in the Nature Portfolio Reporting Summary linked to this article.

## Supplementary information

Supplementary Information

Peer Review File

Description of Additional Supplementary Information

Supplementary Movie 1

Supplementary Movie 2

Supplementary Movie 3

Supplementary Movie 4

Supplementary Movie 5

Supplementary Movie 6

Reporting Summary

## Data Availability

The data that support the results of this study are available from the corresponding authors upon request.

## Code availability

All code used to analyze the dataset is available in Zenodo^26^.
