# Supplementary material for: Formation mechanism of high-index faceted Pt-Bi alloy nanoparticles by evaporation-induced growth from metal salts
Source: Nat Commun. 2023 Jun 24;14:3790. doi: 10.1038/s41467-023-39458-6 (PMC10290712; doi:10.1038/s41467-023-39458-6)
Supplement: Supplementary file 1 — Supplementary Information [file 41467_2023_39458_MOESM1_ESM.pdf]

Supplementary Information

Formation mechanism of high-index faceted Pt-Bi alloy nanoparticles by evaporation-induced growth from metal salts

Kunmo Koo, Bo Shen, Sung-Il Baik, Zugang Mao, Paul J. M. Smeets, Ivan Cheuk, Kun He, Roberto dos Reis, Liliang Huang, Zihao Ye, Xiaobing Hu*, Chad A. Mirkin*, and Vinayak P. Dravid*

*Corresponding authors. Email: xbhu@northwestern.edu, chadnano@northwestern.edu, or v-dravid@northwestern.edu

This PDF file includes:

Supplementary Fig. 1 to 13
Supplementary Table 1

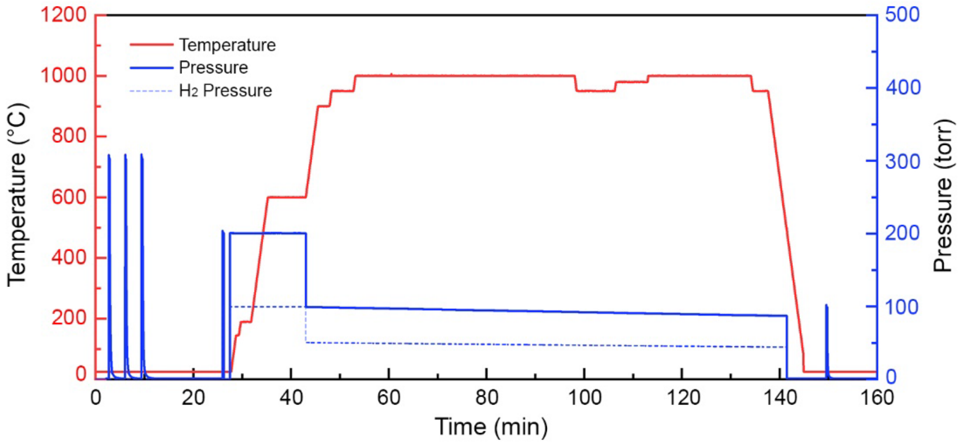

Supplementary Fig. 1. Environmental parameter (pressure and temperature) during an *in situ* GC-TEM experiment. The holder is first purged with 300 torr of UHP argon three times. The initial alloying process is carried out at 600 °C at 200 torr of H_2/Ar mixed gas (flow rate = 0.1 atm cm^3 min^{-1}) for 10 minute*s*. The dealloying process is performed at 1000 °C and 100 torr of H_2/Ar (flow rate = 0.1 atm cm^3 min^{-1}).

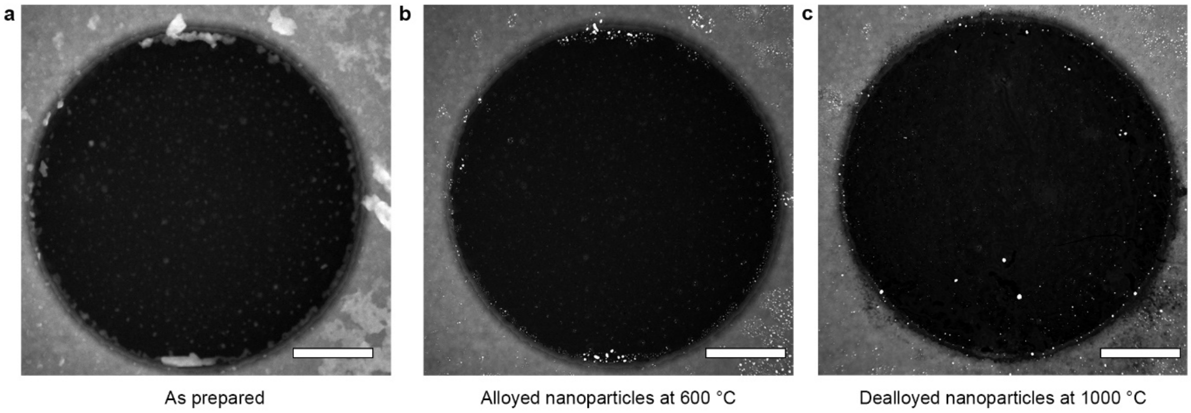

Supplementary Fig. 2. Low-magnification HAADF-STEM images during the *in situ* particle synthesis. **(a)** As-prepared salt precursor before heating, **(b)** after heating the precursor to 600 °C, and **(c)** after coarsening and facet development at 1000 °C. Scale bar = 2 μm.

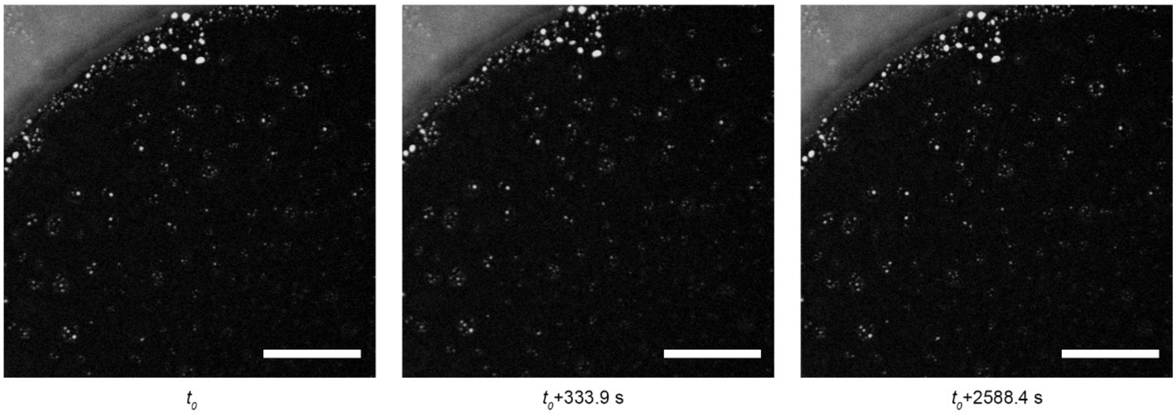

Supplementary Fig. 3. HAADF-STEM images after Pt-Bi alloy formation at 600 °C for extended times. Scale bar = 1 μm.

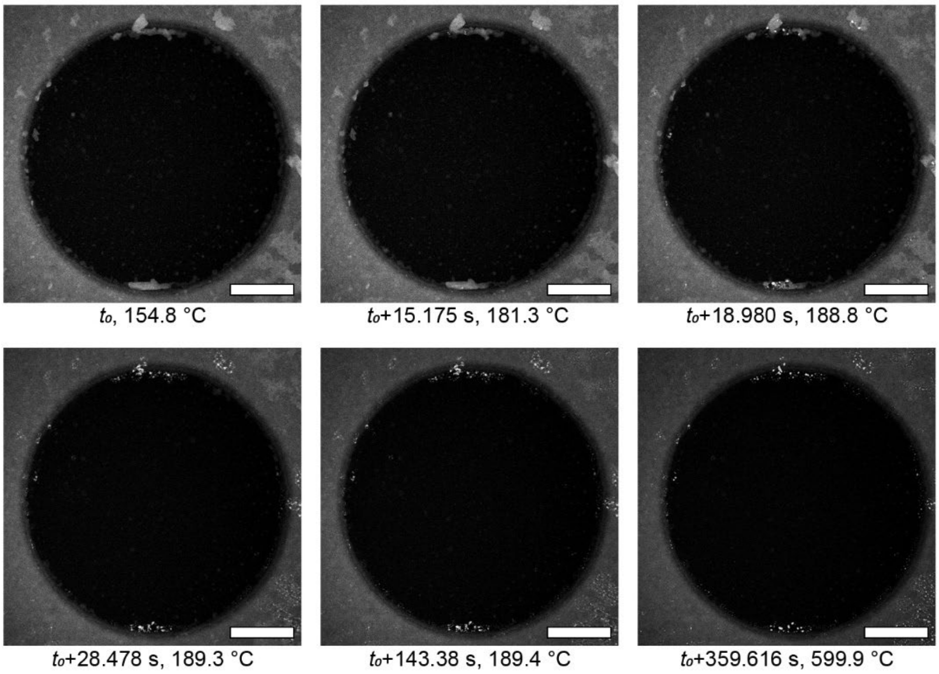

Supplementary Fig. 4. Low-magnification HAADF-STEM images during the alloying stage. Scale bar = 2 μm.

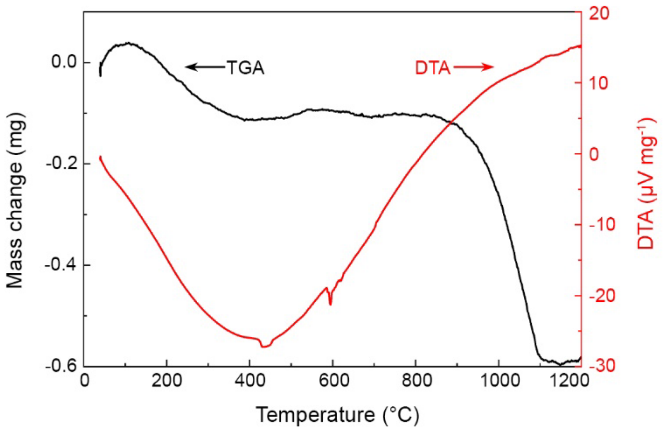

Supplementary Fig. 5. TGA-DTA graph of mixed $H_2PtCl_6{\cdot}6H_2O$ and $BiCl_3$ precursor heated to 1200 °C.

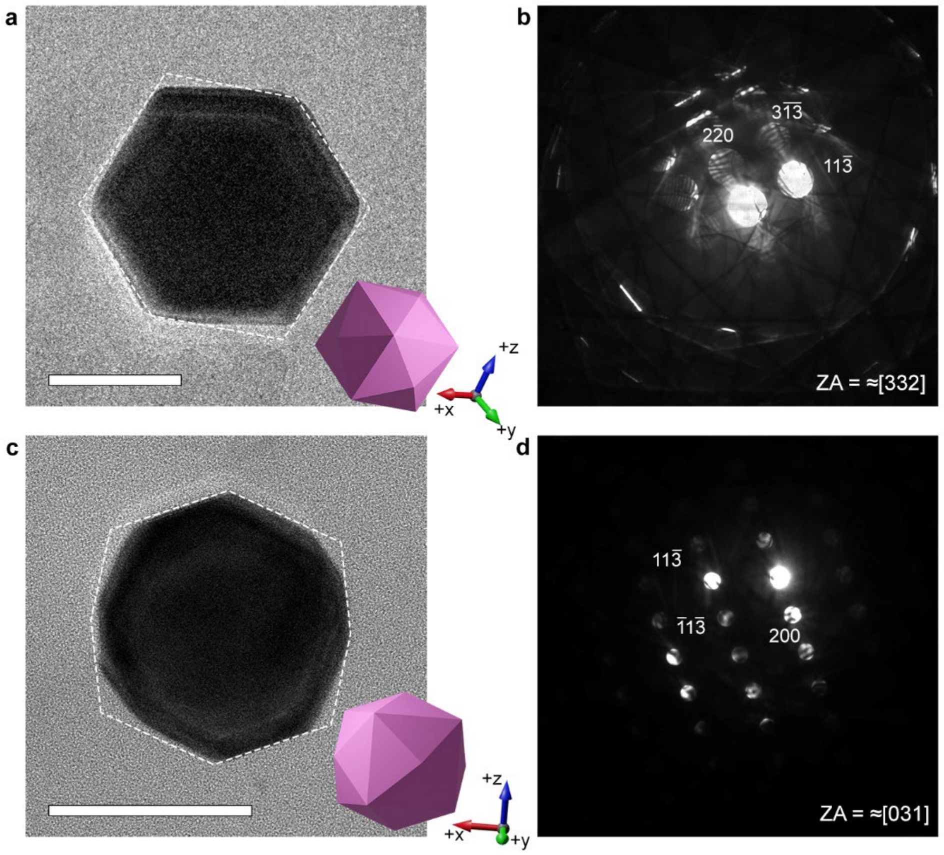

Supplementary Fig. 6. Bright field TEM image and corresponding electron diffraction pattern of an *in situ* synthesized THH Pt nanoparticle. (a, b) THH nanoparticle tilted approximately to the [332] zone-axis. **(c, d)** THH nanoparticle tilted approximately to the [031] zone-axis. The insets and white dotted lines in (a, c) indicate the ideal projection model of a THH shape. The scale bars in (a, c) are 50 nm.

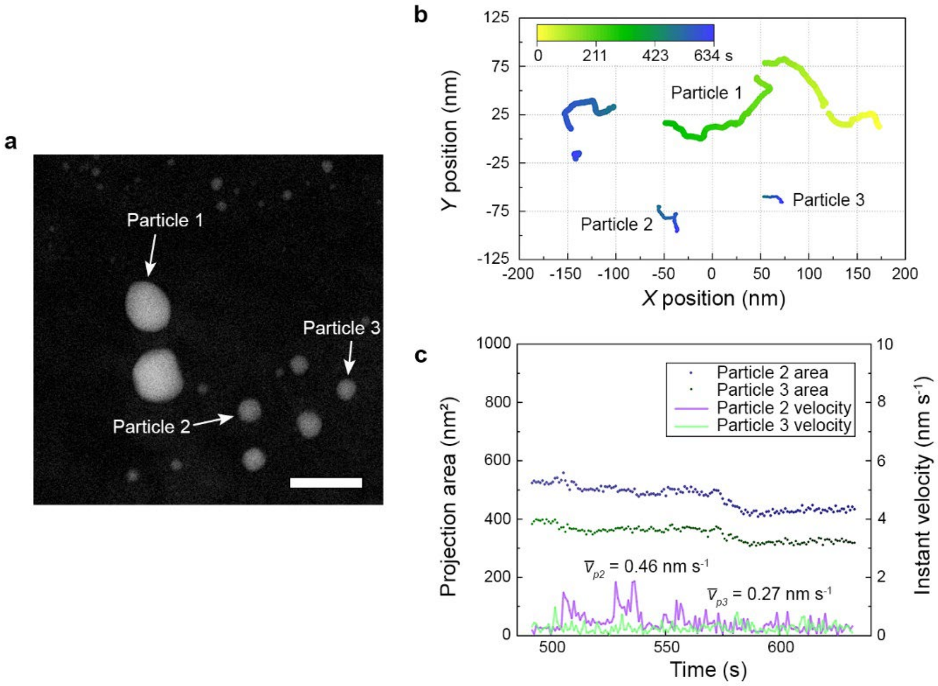

Supplementary Fig. 7. Kinetics analysis of moving particles. (a) HAADF image of tracked nanoparticles. The particles used for kinetic analysis are labeled. The scale bar is 100 nm. **(b)** Moving trajectories of tracked nanoparticles. **(c)** The projection area and instant velocity change of particles 2 and 3. The data for particle 1 is included in the main text (Figure 3).

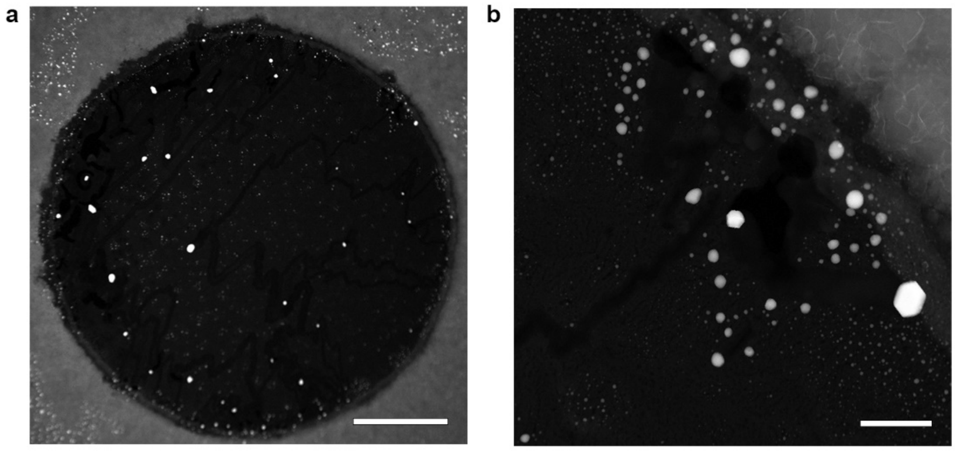

Supplementary Fig. 8. Viewing window without electron beam exposure upon *in situ* THH Pt nanoparticles formation process. (a) Low mag HAADF image. Scale bar = 2 μm. **(b)** Higher magnification HAADF image. Scale bar = 200 nm.

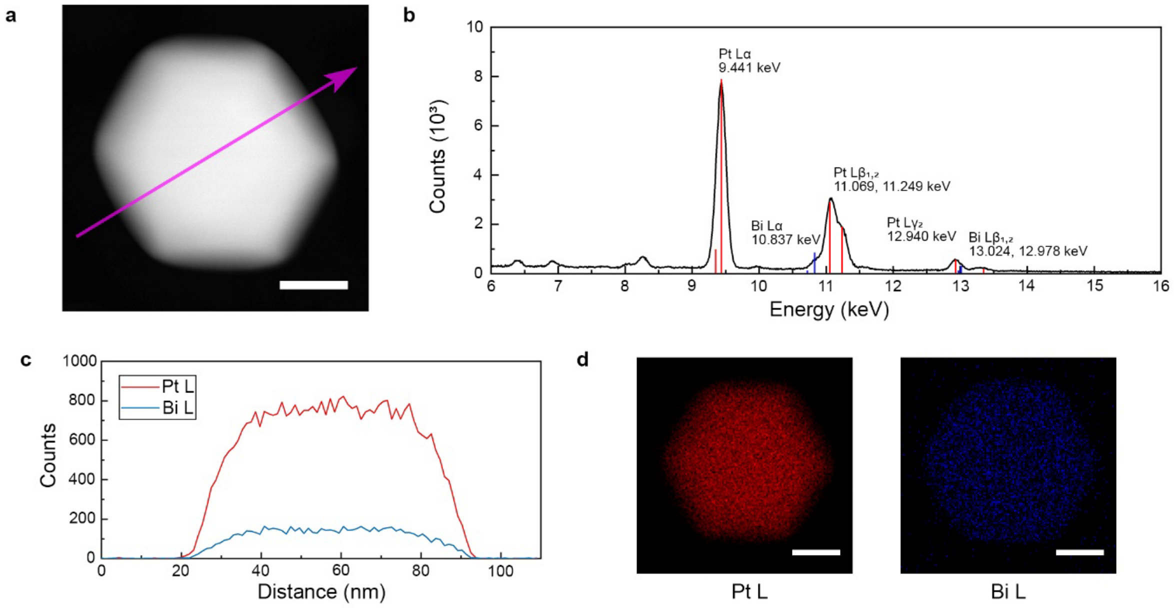

Supplementary Fig. 9. **STEM-EDS analysis of a THH-faceted Pt nanoparticle. (a)** HAADF-STEM image of the nanoparticle projected along ≈ [110] direction. Scale bar = 20 nm. **(b)** EDS profiles showing the overlapping Pt L and Bi L. **(c)** Line profile of Pt L and Bi L across the arrowed line in (a). **(d)** Elemental map corresponding to the nanoparticle in **(a)**. Scale bar = 20 nm.

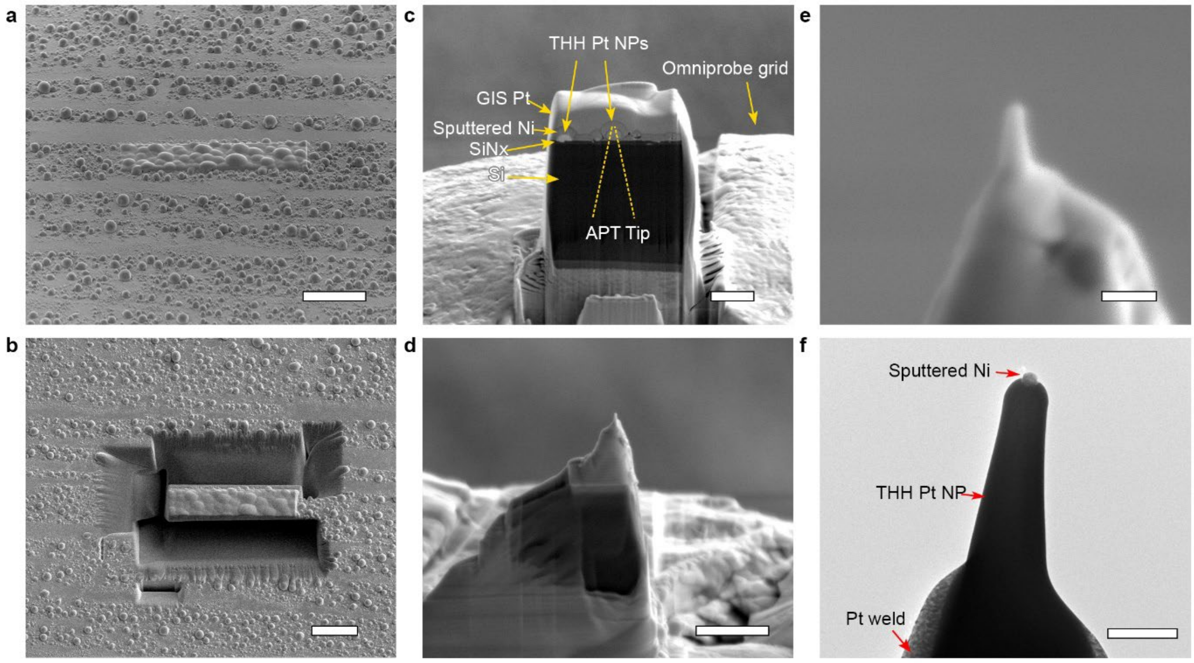

Supplementary Fig. 10. Preparation steps of APT tips of THH Pt nanoparticles. (a) Deposition of Pt-protection layer on Ni-coated particles. Scale bar = 10 μm. **(b)** Lift-out process. Scale bar = 10 μm. **(c)** Welding of the lift-out lamina to the Omniprobe grid. Scale bar = 2 μm. **(d)** A sharpened APT tip. Scale bar = 2 μm. **(e)** A close-up ion beam image of an APT tip. Scale bar = 200 nm. **(f)** A bright-field TEM image of an APT sample. Scale bar = 100 nm.

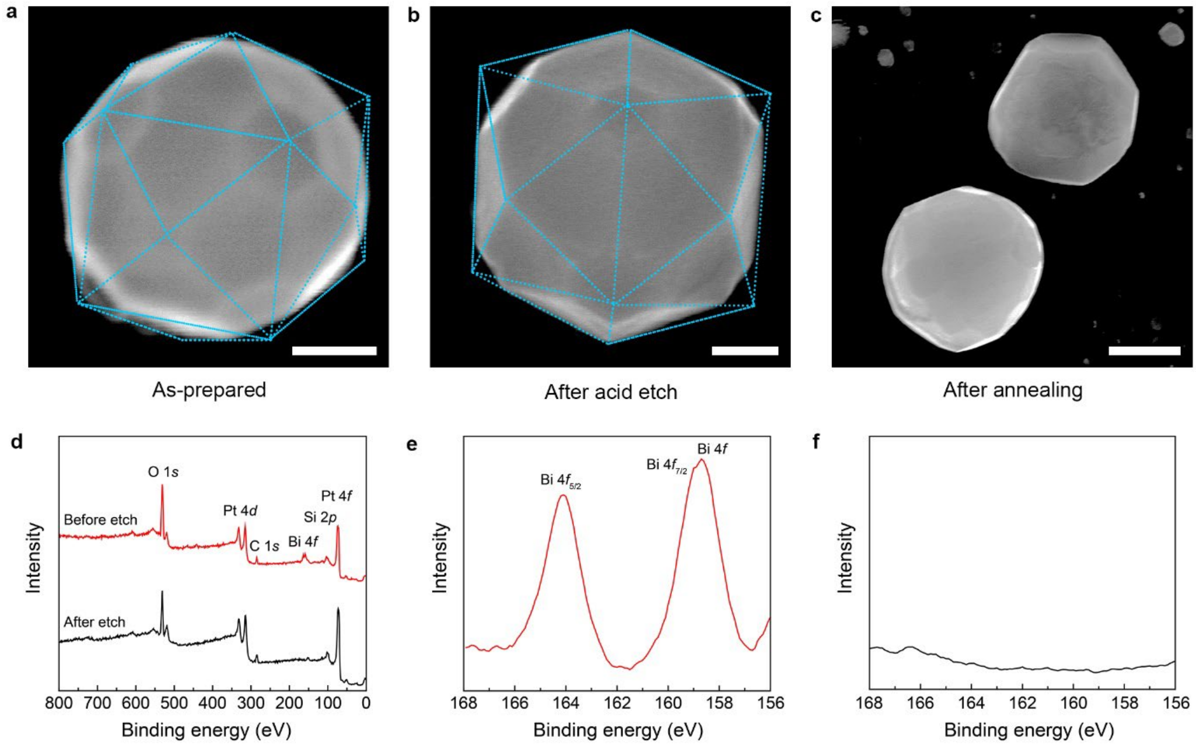

Supplementary Fig. 11. Stable shapes of Pt nanoparticles before and after removing surficial Bi atoms (a) As synthesized THH Pt nanoparticle with Bi segregation at surfaces. Scale bar = 100 nm. **(b)** THH Pt nanoparticle after soaking to 1 M H_2SO_4 for 1 h to remove surface Bi. The cyan colored dotted guideline indicates the ideal (210) facets. Scale bar = 100 nm. **(c)** Thermally annealed (1000 °C, 10 min) THH Pt nanoparticles after acid etching. Scale bar = 200 nm. **(d-f)** XPS survey spectra of THH Pt nanoparticles before (red) and after acid etch (black). XPS Bi 4*f* spectra of THH nanoparticles (e) before and (f) after acid etching.

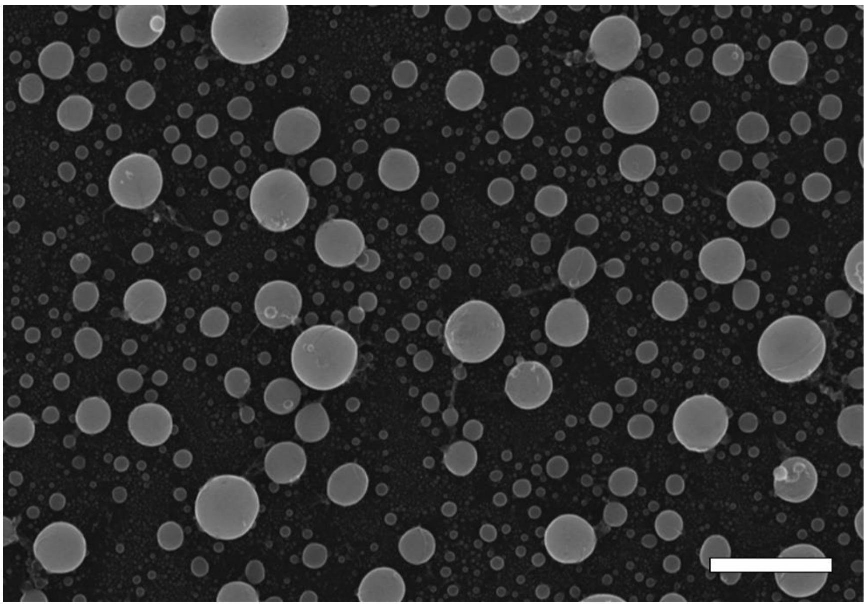

Supplementary Fig. 12. Pt nanoparticles after 3 h annealing at 1000 °C. Scale bar = 1 μm.

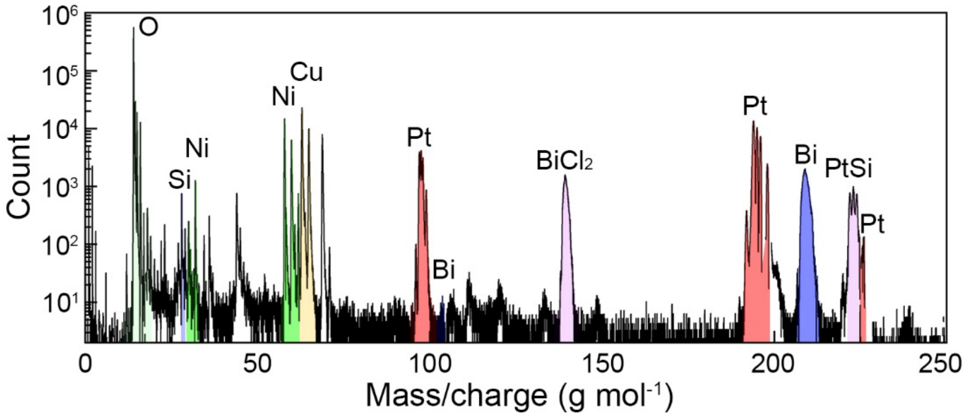

Supplementary Fig. 13. Mass spectrum of the APT sample. The color fill indicates the peak integration area for quantifying elements (i.e., Figure 6).

	No Bi	Homogeneous Pt/Bi alloy	Bi at surface (25%)	Bi at surface (50%)	Bi at surface (75%)	Bi at surface (100%)
(100)	1.712	1.452	0.974	1.228	1.664	3.932
(110)	2.354	1.665	1.211	0.875	1.781	2.346
(111)	1.395	1.642	2.314	3.858	3.449	8.583
(210)	2.435	2.156	1.154	0.953	0.696	0.465
Average	1.448	1.556	1.020	0.895	0.696	0.465

Supplementary Table 1. Surface energy of several crystallographic planes calculated from DFT. Average surface energy in the last row indicates the surface energy of the finalized Wulff shape at each concentration. The unit of energy is J m^{-2}.
