# Supplementary material for: Formation mechanism of high-index faceted Pt-Bi alloy nanoparticles by evaporation-induced growth from metal salts
Source: Nat Commun. 2023 Jun 24;14:3790. doi: 10.1038/s41467-023-39458-6 (PMC10290712; doi:10.1038/s41467-023-39458-6)
Supplement: Supplementary file 3 — Description of Additional Supplementary Information [file 41467_2023_39458_MOESM3_ESM.docx]

Supplementary Movie 1. Nucleation of Pt-Bi alloyed seeds from the salt precursors

Supplementary Movie 2. Coalescence growth of Pt-Bi alloy nanoparticle seeds

Supplementary Movie 3. Facet development of THH Pt Nanoparticle direction

Supplementary Movie 4. Facet development of another THH Pt Nanoparticle

Supplementary Movie 5. Atom probe tomography of THH Pt nanoparticle

Supplementary Movie 6. Dynamic tracking of nanoparticles
