# Supplementary material for: Formation mechanism of high-index faceted Pt-Bi alloy nanoparticles by evaporation-induced growth from metal salts
Source: Nat Commun. 2023 Jun 24;14:3790. doi: 10.1038/s41467-023-39458-6 (PMC10290712; doi:10.1038/s41467-023-39458-6)
Supplement: Supplementary file 2 — Peer Review File [file 41467_2023_39458_MOESM2_ESM.pdf]

nature portfolio

# Peer Review File

## Formation mechanism of high-index faceted Pt-Bi alloy nanoparticles by evaporation-induced growth from metal salts

**Open Access** This file is licensed under a Creative Commons Attribution 4.0 International License, which permits use, sharing, adaptation, distribution and reproduction in any medium or format, as long as you give appropriate credit to the original author(s) and the source, provide a link to the Creative Commons license, and indicate if changes were made. In the cases where the authors are anonymous, such as is the case for the reports of anonymous peer reviewers, author attribution should be to 'Anonymous Referee' followed by a clear attribution to the source work. The images or other third party material in this file are included in the article's Creative Commons license, unless indicated otherwise in a credit line to the material. If material is not included in the article's Creative Commons license and your intended use is not permitted by statutory regulation or exceeds the permitted use, you will need to obtain permission directly from the copyright holder. To view a copy of this license, visit http://creativecommons.org/licenses/by/4.0/.

**REVIEWER COMMENTS**

Reviewer #1 (Remarks to the Author):

In this work, the authors utilized in situ gas-cell transmission electron microscopy (GC-TEM) to investigate the formation mechanism of high-index-faceted Pt nanoparticles in the presence of volatile foreign metal (Bi). They also employed atom probe tomography (APT) and density functional theory (DFT) calculations to uncover the underlying mechanism of the alloying-dealloying process of Bi. The findings presented in this manuscript are interesting and can provide insights into the formation of high-index-faceted nanoparticles achieved by volatile elements. Therefore, I recommend the publication of this work in this journal. Followings are some comments that should be addressed before publication.

1) Could the authors provide a more convincing experimental evidence that supports the claim that the stable structure changes depending on the degree of surface segregation of Bi atoms? Specifically, I wonder whether the change in structure is due to a change in the degree of surface segregation of Bi atoms.

2) Discussion on why the resultant nanoparticles take specifically a truncated THH structure rather than other morphologies enclosed by high-index facets should be added.

3) There is no explanation on the possible influences of the electron beam in the in situ TEM experiments, although it is expected to have a significant impact.

4) What happens if the THH nanoparticles are exposed to 1000 ℃ for a prolonged time?

Reviewer #2 (Remarks to the Author):

In the work of Koo et al., by using in situ gas cell TEM, the authors studied the transformation of Pt salts to spherical seeds and then to tetrahexahedra, in the presence of trace Bi. Their results and conclusions are further supported by DFT calculations and atom probe tomography experiments. I find this work excellent and comprehensive and the results will be of interest to a broad scientific audience. Therefore I strongly recommend its publication in Nature Communications. I have only one minor comment related to the presence of Bi on the final particles.
In the introduction of the draft, it is mentioned that tetrahexahedral (THH) Pt nanoparticles exhibit excellent activity as catalysts in formic acid oxidation compared with their low-index facet counterparts, due, in part, to the high density of surface atoms with low coordination numbers. Since on the surface of the final particles of this work traces of Bi are expected, could the authors comment on the effect this would have on the catalytic activity towards specific applications?

Reviewer #3 (Remarks to the Author):

This manuscript includes nice experimental results for the synthesis of tetrahexahedral Pt nanoparticles. It is very interesting for the authors to successfully synthesize high-index facet Pt particles by using Pt/Bi alloying-dealloying processes. The authors performed a series of combined analysis, such as in-situ TEM, APT, and DFT calculations, to explain the role of Bi in forming high-index facet Pt particles. I would like to make two important questions or suggestions.

1. The authors present calculated surface energies (Table S1) for Pt/Bi alloys without presenting the detailed surface model configurations and equations used for the values presented. I am wondering if it is really possible to calculate surface energies for certain facets of alloy surfaces. The lack of a description of detailed computational approaches does not match the importance of the experimental

results.

2. In line 60, the authors suggest that the basic understanding achieved in this work can be applied to "other metallic systems". However, the authors did not present results for other metallic systems. How can we accept that the synthesis principle introduced by the authors can be directly applied to other metallic systems?

## Reviewer #1

**Remarks to the Authors**:

In this work, the authors utilized in situ gas-cell transmission electron microscopy (GC-TEM) to investigate the formation mechanism of high-index-faceted Pt nanoparticles in the presence of volatile foreign metal (Bi). They also employed atom probe tomography (APT) and density functional theory (DFT) calculations to uncover the underlying mechanism of the alloying-dealloying process of Bi. The findings presented in this manuscript are interesting and can provide insights into the formation of high-index-faceted nanoparticles achieved by volatile elements. Therefore, I recommend the publication of this work in this journal. The following are some comments that should be addressed before publication.

Response: Thank the reviewer for the recommendation and raised points which are helpful for us to enhance the quality of our work. The reviewer's comments have been addressed as follows.

1. Could the authors provide a more convincing experimental evidence that supports the claim that the stable structure changes depending on the degree of surface segregation of Bi atoms? Specifically, I wonder whether the change in structure is due to a change in the degree of surface segregation of Bi atoms.

Response: We thank the reviewer for the constructive comments. We acknowledge that the analysis of the localized Bi within the THH nanoparticle is extremely important. Thus, we used APT to characterize the Bi distribution in the Pt particles. However, as shown in our video, the shape evolution process of Pt nanoparticles is fast, and it is very challenging to capture the intermediate state with 25% or 50% Bi segregation for APT characterization. To demonstrate the role of the surface segregation of Bi atoms to the stable structure, we removed the surface Bi atoms of THH Pt particles by acid etching (1 M $H_2SO_4$ for 1 h), and then annealed the etched Pt nanoparticle at 1000 °C for 10 min to obverse the shape change. The result shows that without Bi, the (210) facets were not stable and disappeared after 10 min annealing. We have incorporated this result into the main text for comprehensive understanding to the readers.

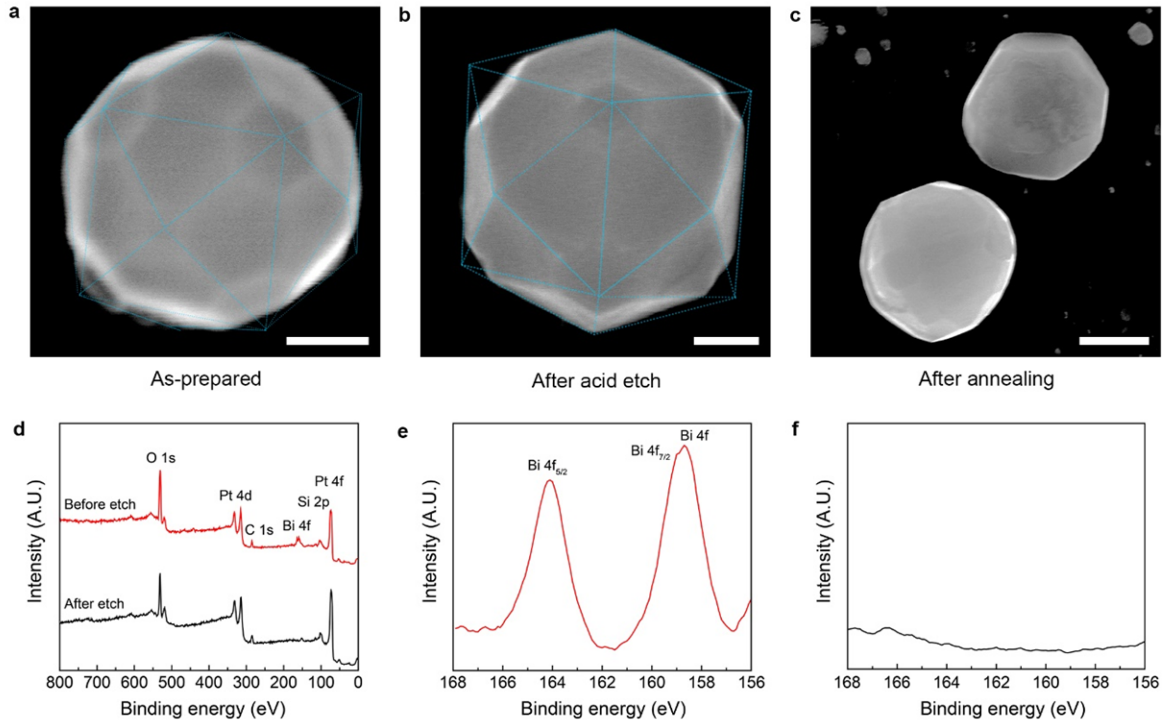


**Supplementary Fig. 11. Stable shapes of Pt nanoparticles before and after removing surficial Bi atoms (A)** As synthesized THH Pt nanoparticle with Bi segregation at surfaces. Scale bar = 100 nm. **(B)** THH Pt nanoparticle after soaking to 1 M $H_2SO_4$ for 1 h to remove surface Bi. The cyan colored dotted guideline indicates the ideal (210) facets. Scale bar = 100 nm. **(C)** Thermally annealed (1000 °C, 10 min) THH Pt nanoparticles after acid etching. Scale bar = 200 nm. **(D-F)** XPS survey spectra of THH Pt nanoparticles before (red) and after acid etch (black). XPS Bi 4f spectra of THH nanoparticles **(E)** before and **(F)** after acid etching.

2. Discussion on why the resultant nanoparticles take specifically a truncated THH structure rather than other morphologies enclosed by high-index facets should be added.

Response: In our previous work (ref. 10, *Science* **2019**, *365*, 1159-1163), we calculated surface energies of different high-index facets in Pt nanoparticles with 100% surficial Bi coverage, including (210), (211), (221), (310) and (320) facets, and the results show that the (210) facets own the lowest surface energy among all the high-index facets evaluated. Hence, the stable morphology of Pt particles is tetrahexahedron with (210) facets instead of other morphologies. Thus, the aforementioned faceting naturally results in the morphology of nanoparticles consistence with truncated THH structure. Additionally, particles tend to present a truncated structure to

decrease the surface areas, which has been widely observed in octahedron shaped nanoparticles (ref. 23, *Angew. Chem. Int. Ed.*, **2009**, *48*, 60-103).

3. There is no explanation on the possible influences of the electron beam in the in situ TEM experiments, although it is expected to have a significant impact.

Response: We agree with the reviewers that the influences of the electron beam should be carefully evaluated for all TEM experiments. But during the *in situ* TEM observations, we found that the effect of electron beam is negligible. **Supplementary Fig. 8** is additional HAADF-STEM images at low magnification, which was obtained from the electron beam-free, adjacent area experiencing *in situ* THH formation process. Within the viewing window (10 $\mu m$ diameter), the coarsening is evenly but randomly occurred throughout the area. At higher magnification, we can still see many faceted nanoparticles This is a strong indication that our Bi segregation and facet regulations are independent to the electron exposure.

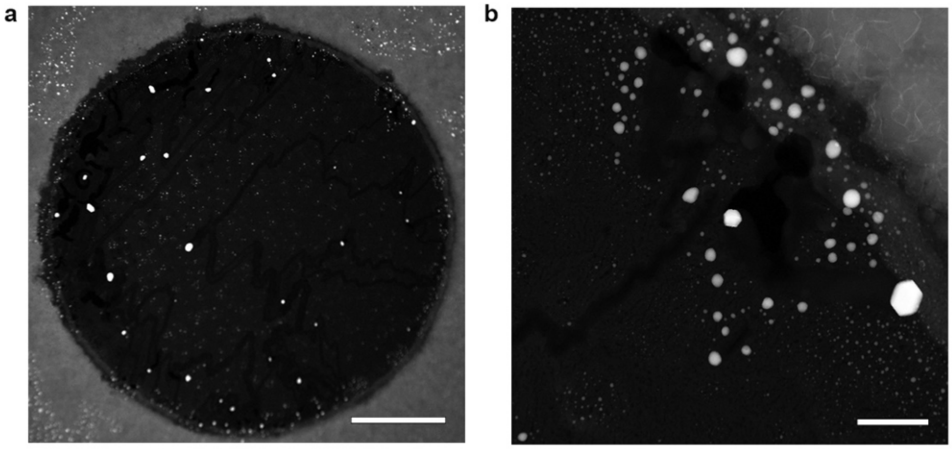


**Supplementary Fig. 8. Viewing window without electron beam exposure upon *in situ* THH Pt nanoparticles formation process. (A)** Low mag HAADF image. Scale bar = 2 µm. **(B)** Higher magnification HAADF image. Scale bar = 200 nm.

There are few reasons that the electron beam effect plays negligible role upon the in situ observation. Normally, the possible electron beam effect can fall into several categories – knock-on damage, radiolysis, charging, and thermal damage (*Microscopy Today* **2021**, *29*, 56-59).

First, knock-on damage can be excluded since this process is only applicable to light, carbonaceous materials but inefficient to dissociate the metals (Bi/Pt) and semiconductor material ($SiN_x$).

Second, for the radiolysis damage, surrounding environmental gas ($H_2$/Ar) can be ionized from the electron radiation. However, radiolysis effect of environmental gases is negligible due to two reasons: the atomic concentration at gas is significantly small (0.04 M) for standard temperature and pressure (STP) Ar compared to the liquid (55.6 M for water). The diffusivity of the radicals is extremely fast ($10^5$-$10^6$ times faster than liquid at STP) and this diffusivity is even higher at temperature during the reaction process. Therefore, the possible electron-beam dissociated species cannot be built up to a meaningful concentration.

Third, for the charging effect, Pt, Bi, and their alloys are metallic and highly conductive ($9.4 \times 10^6$ S $m^{-1}$) so that localized charge imbalance should not occur, but only $SiN_x$ membrane can slightly suffer from charging.

Lastly, for the heating, electron irradiation does elevate the sample temperature up from 10 K (*Microscopy Today* **2021**, *29*, 56-59) to 20 K (*Adv. Sci.* **2020**, *8*, 2002876) depending on the sample thickness and thermal conductivity. But even in the extreme case, it can be disregarded since our reaction temperature is already 1273 K (only 0.8-1.6% contribution).

4. What happens if the THH nanoparticles are exposed to 1000 ℃ for a prolonged time?

Response: A prolonged period (>3 h) of annealing, beyond our *in situ* observation timescale, at 1000 °C will eventually destroy the THH shape due to the loss of Bi (**Supplementary Fig. 12**). Compared to Figure 1, the resulting annealed particle loses all characteristic facets and becomes a spherical shape. We have added to the result section for the comprehensive understanding to the readers.

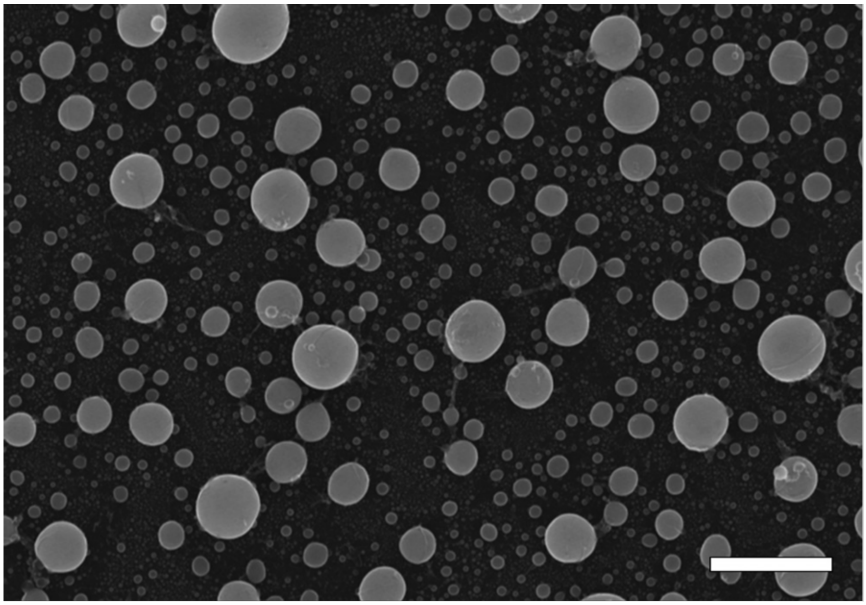

**Supplementary Fig. 12. Pt nanoparticles after 3 *h* annealing at 1000 °*C*.** Scale bar = 1 *μm*.

## Reviewer #2

**Remarks to the Authors**:

In the work of Koo et al., by using in situ gas cell TEM, the authors studied the transformation of Pt salts to spherical seeds and then to tetrahexahedra, in the presence of trace Bi. Their results and conclusions are further supported by DFT calculations and atom probe tomography experiments. I find this work excellent and comprehensive and the results will be of interest to a broad scientific audience. Therefore I strongly recommend its publication in Nature Communications. I have only one minor comment related to the presence of Bi on the final particles.

In the introduction of the draft, it is mentioned that tetrahexahedral (THH) Pt nanoparticles exhibit excellent activity as catalysts in formic acid oxidation compared with their low-index facet counterparts, due, in part, to the high density of surface atoms with low coordination numbers. Since on the surface of the final particles of this work traces of Bi are expected, could the authors comment on the effect this would have on the catalytic activity towards specific applications?

Response: We thank the reviewer for the comments. Indeed, the surface Bi can influence the catalytic properties in specific reactions. We have previously explored the contributions of the surficial Bi to activity enhancement toward the electrooxidation of formic acid (ref. 12, *Adv. Mater.* **2020**, *32*, 2002849). We evaluated the contributions of surficial Bi and high-index facets

individually. The results showed that the activity of Pt/C with surficial Bi exhibited ~17 times higher than that of the commercial Pt/C catalyst. On the other hand, the activity of THH Pt nanocatalysts (with surficial Bi) showed ~4 times higher than that of Pt/C with surficial Bi and ~68 times higher than that of the commercial Pt/C catalyst. Hence, both surficial Bi and THH shape can promote electrooxidation of formic acid.

## Reviewer #3

**Remarks to the Authors**:

This manuscript includes nice experimental results for the synthesis of tetrahexahedral Pt nanoparticles. It is very interesting for the authors to successfully synthesize high-index facet Pt particles by using Pt/Bi alloying-dealloying processes. The authors performed a series of combined analysis, such as in-situ TEM, APT, and DFT calculations, to explain the role of Bi in forming high-index facet Pt particles. I would like to make two important questions or suggestions.

Response: Thank the reviewer for the compliments on our work and raised suggestions. We have modified our manuscript to reflect these suggestions.

1. The authors present calculated surface energies (Table S1) for Pt/Bi alloys without presenting the detailed surface model configurations and equations used for the values presented. I am wondering if it is really possible to calculate surface energies for certain facets of alloy surfaces. The lack of a description of detailed computational approaches does not match the importance of the experimental results.

Response: Thank you for the constructive comments. We added the following details about the model configurations and equations used.

The summation over the Brillouin zone for the bulk structures was performed on an 8 × 8 × 8 Monkhorst-pack k-point mesh for all calculations. Bulk $Pt_{108-n}Bi_n$ with and without vacuum separation were constructed for different surface planes. To simulate the alloying process, Bi atoms are randomly substituted for the interior Pt atoms. For models with 25%, 50%, 75% and 100% surface coverages in the dealloying process, we force 25%, 50%, 75% and 100% Bi atoms to

substitute the Pt atoms on the surface. The total energies with volume constraint were calculated for these fully relaxed model structures. The surface energy, σ, of different planes is determined by the following:

$$\sigma = (E_{Surface}^{bulkPt_{108-n}Bi_n} - E^{bulkPt_{108-n}Bi_n})/A$$

Where $E_{Surface}^{bulkPt_{108-n}Bi_n}$ is the total bulk energy of Pt108-nBin of different surface planes with 6 Å vacuum separation, $E^{bulkPt_{108-n}Bi_n}$ is the total bulk energy of Pt108-nBin with no surface, and A is the area of the surface. The substituting energy of Bi in Pt bulk is 0.756 eV atom-1. The surface was built on an 8 × 8 × 2 slab with a 6 Å vacuum. We have found that 6 Å plane separation is sufficient to minimize the effects between separation planes. Wulff construction was used to predict the morphology of the nanoparticles.

2. In line 60, the authors suggest that the basic understanding achieved in this work can be applied to "other metallic systems". However, the authors did not present results for other metallic systems. How can we accept that the synthesis principle introduced by the authors can be directly applied to other metallic systems?

Response: This is an outlook of this alloying-dealloying method. In our previous work, we have prepared a library of THH shaped mono- and multi-metallic nanoparticles with face-centered cubic (*fcc*) structures including Co, Ni, Rh, Pd and Pt (ref. 10, *Science* **2019**, *365*, 1159-1163 and ref. 13, *PNAS*, **2019**, *118*, e2105722118). Because all THH shaped nanoparticles are obtained via alloying-dealloying process (the same as Pt-Bi system), this work can be used as a general explanation to the THH particle formation mechanism. We have added the ref. 10 and ref. 13 in the main text. Additionally, we have prepared high-index facet, *hcp*-structured Ru particles shown in the following figure and we are working on analyzing the facets, which would be in our future work.

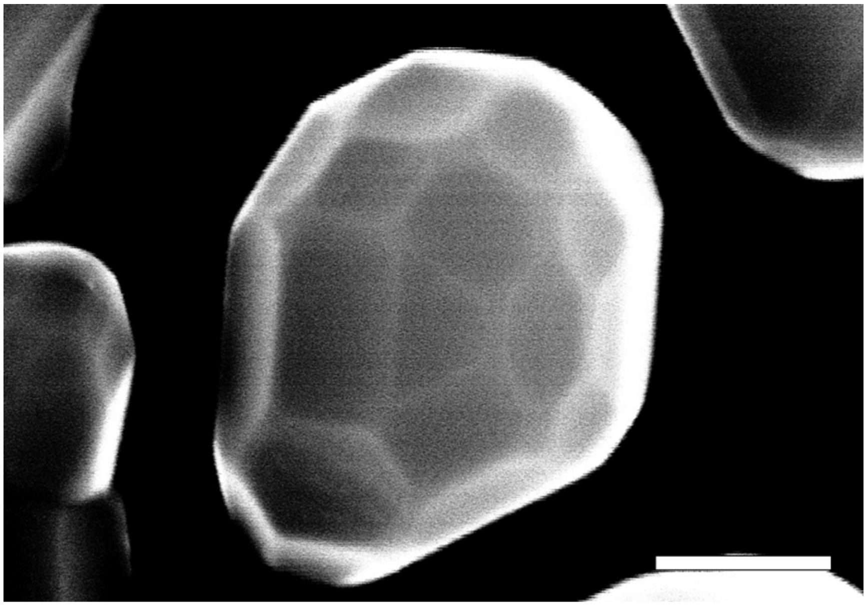

**Figure of Ru nanoparticles with high-index facets.** The scale bar is 200 nm.

**REVIEWERS' COMMENTS**

Reviewer #1 (Remarks to the Author):

The authors carefully responded to my comments and revised the manuscript well. I recommend the publication of this paper.

Reviewer #2 (Remarks to the Author):

The authors addressed my previous comments . I am satisfied with the extra work they did and have no further comments from my side.
